# Educational simulator for mastoidectomy considering mechanical properties using 3D printing and its usability evaluation

**DOI:** 10.1038/s41598-024-58359-2

**Published:** 2024-04-01

**Authors:** Junhyeok Ock, Yeonjoo Choi, Dong-Gyu Lee, Jong Woo Chung, Namkug Kim

**Affiliations:** 1 Department of Convergence Medicine, Asan Medical Institute of Convergence Science and Technology, University of Ulsan College of Medicine, Asan Medical Center, 88 Olympic-Ro 43-Gil Songpa-Gu, Seoul, 05505 South Korea; 2 Department of Otorhinolaryngology-Head & Neck Surgery, University of Ulsan College of Medicine, Asan Medical Center, 88 Olympic-Ro 43-Gil Songpa-Gu, Seoul, 05505 South Korea; 3 Department of Radiology, University of Ulsan College of Medicine, Asan Medical Center, 88 Olympic-Ro 43-Gil Songpa-Gu, Seoul, 05505 South Korea

**Keywords:** Three-dimensional (3D) printing, Computed tomography (CT), Educational simulator, Insertion torque, Mastoidectomy, Mechanical properties, Shape accuracy, Anatomy, Engineering

## Abstract

Complex temporal bone anatomy complicates operations; thus, surgeons must engage in practice to mitigate risks, improving patient safety and outcomes. However, existing training methods often involve prohibitive costs and ethical problems. Therefore, we developed an educational mastoidectomy simulator, considering mechanical properties using 3D printing. The mastoidectomy simulator was modeled on computed tomography images of a patient undergoing a mastoidectomy. Infill was modeled for each anatomical part to provide a realistic drilling sensation. Bone and other anatomies appear in assorted colors to enhance the simulator’s educational utility. The mechanical properties of the simulator were evaluated by measuring the screw insertion torque for infill specimens and cadaveric temporal bones and investigating its usability with a five-point Likert-scale questionnaire completed by five otolaryngologists. The maximum insertion torque values of the sigmoid sinus, tegmen, and semicircular canal were 1.08 ± 0.62, 0.44 ± 0.42, and 1.54 ± 0.43 N mm, displaying similar-strength infill specimens of 40%, 30%, and 50%. Otolaryngologists evaluated the quality and usability at 4.25 ± 0.81 and 4.53 ± 0.62. The mastoidectomy simulator could provide realistic bone drilling feedback for educational mastoidectomy training while reinforcing skills and comprehension of anatomical structures.

## Introduction

The temporal bone, with its intricate anatomical structures, poses technical challenges during mastoidectomy operations, elevating the risk factor for patients^1^. To pre-emptively mitigate these risks, surgeons must continually hone their skills via diverse training methods. Skill training ensures patient safety and yields improved surgical outcomes^2,3^. Traditional surgical skill training has been predominantly based on apprenticeship; however, with advancements, such as minimally invasive surgery, robotic surgery, and other technological developments, this approach faces limitations^4^. Consequently, the surgical technique training method has expanded to include box trainers, animal models, cadavers, virtual reality devices, and full procedural simulators^2^.

However, the use of animal models and cadavers is challenged by problems like cost, accessibility, and ethics. In addition, the declining number of bodies donated for research further compounds this problem^5–7^. Virtual reality devices have obstacles, such as a lack of realistic simulation of drill feedback, high initial setup costs, and concerns regarding patient data anonymization^8^. High-fidelity simulators are associated with considerable expense^9^.

In addition, three-dimensional (3D) printing technology has the advantage of readily fabricating complex shapes; therefore, it has been applied to medical healthcare in various areas, such as surgical guides, implants, and simulators^10^. A growing number of research groups are exploring the fabrication of mastoidectomy simulators using 3D printing due to their advantages in forming intricate shapes and their cost-effectiveness^11–14^. However, problems persist regarding the lack of reproducibility regarding each anatomical structure and the quantitative analysis of the temporal bone and evaluations, which are solely qualitative and depend on surgeons’ surveys.

To overcome these problems, we developed and evaluated a realistic mastoidectomy simulator considering mechanical properties using 3D printing based on computed tomography (CT) images by comparing the screw insertion torque of various infill specimens and cadavers. In addition, surgeon satisfaction was evaluated through a questionnaire survey.

## Methods

**Figure 1:**
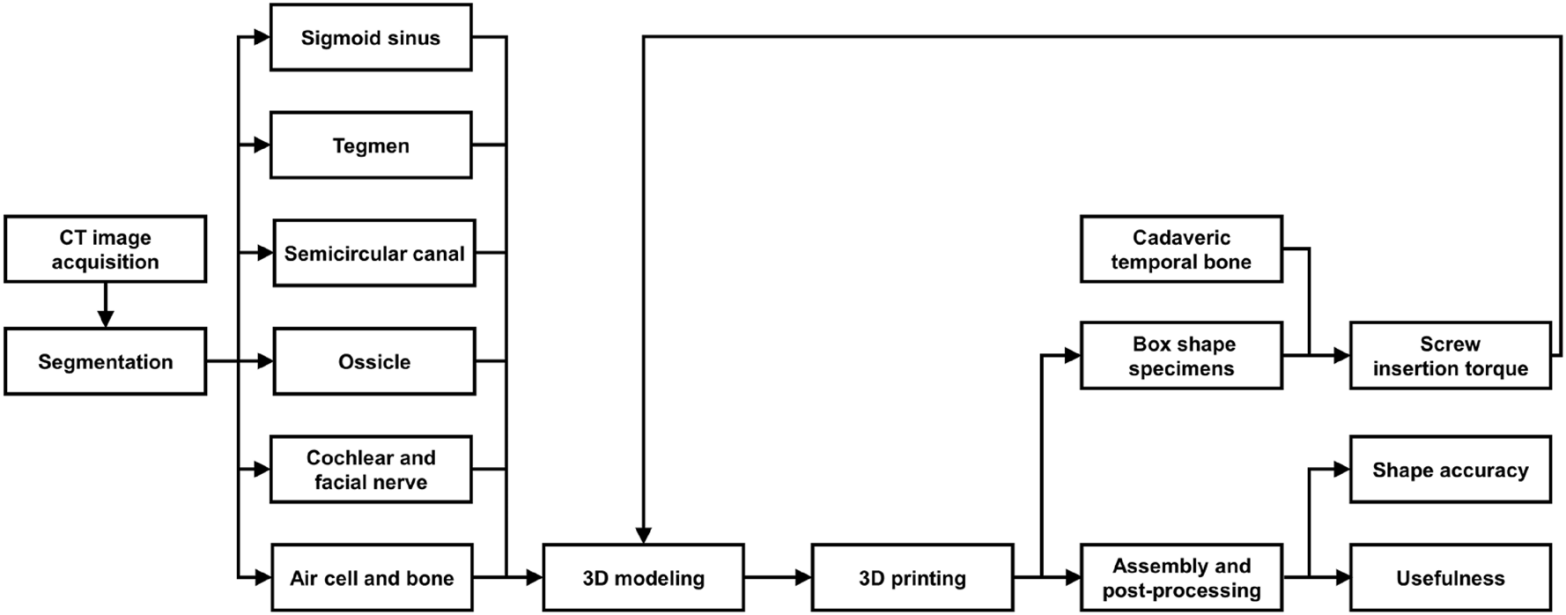
Overall procedure of fabricating and evaluating a mastoidectomy simulator (CT: computed tomography).

Figure 1 illustrates the overall process of fabricating and evaluating a mastoidectomy simulator. First, various anatomical structures necessary for the mastoidectomy simulator were semi-automatically segmented and modeled based on the CT image of a patient. Each anatomical structure was modeled and fabricated according to the mechanical properties and color.

An otolaryngologist measured and compared the screw insertion torque using various infill specimens and a cadaver. The fabricated anatomical structure was assembled into a single phantom and evaluated by measuring the shape accuracy and assessing the quality and validity through a survey completed by the otolaryngologist.

### Computed tomography image acquisition and segmentation

This study was approved by the Institutional Review Board of the Asan Medical Center (IRB No. 2023-0292) and was performed in accordance with the principles of the Declaration of Helsinki. The requirement for informed consent was waived due to the retrospective nature of this study without additional harm to the patient.

**Figure 2:**
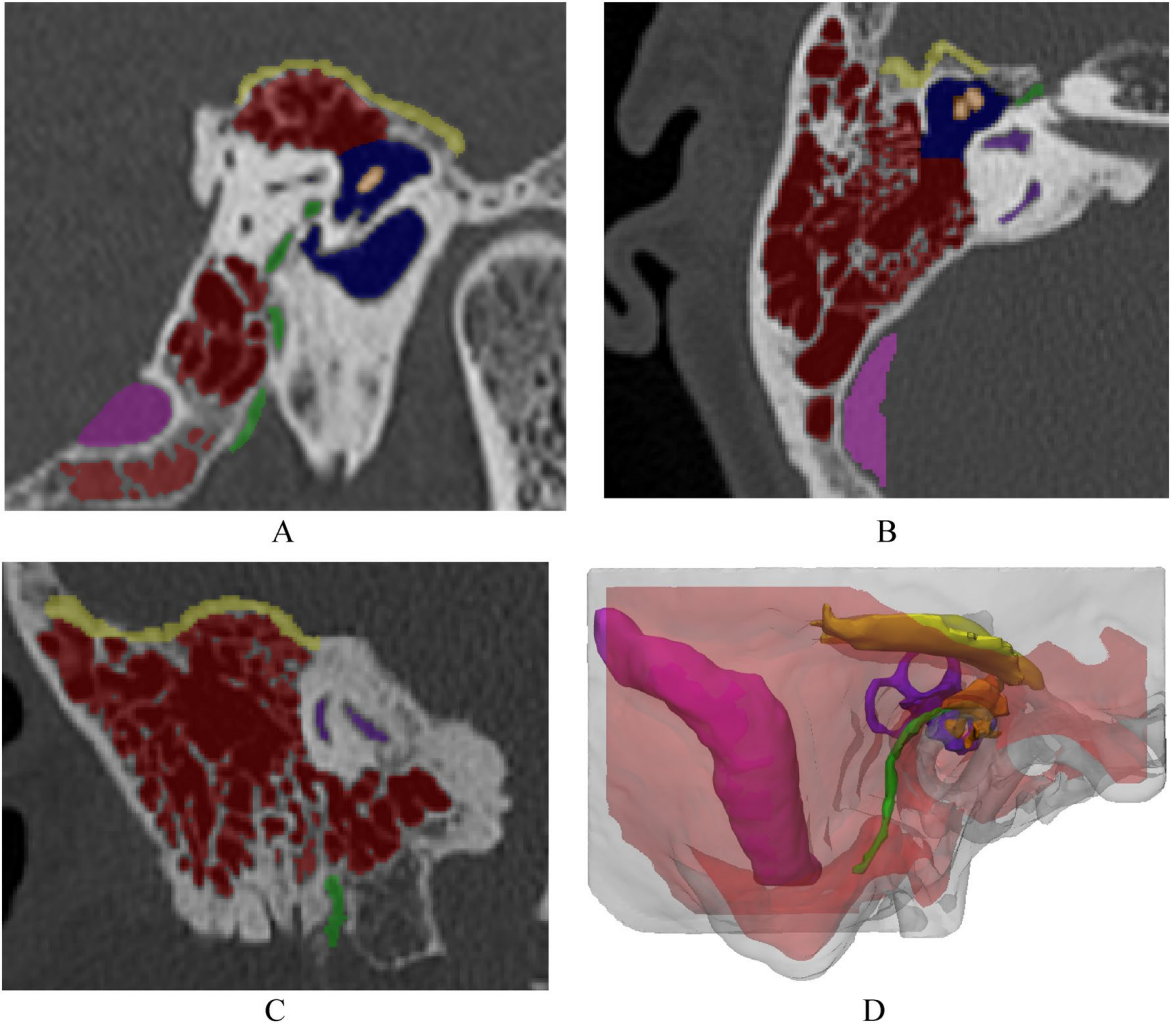
Visualization of the anatomical structures for modeling the mastoidectomy simulator based on computed tomography images: (**A**) sagittal view, (**B**) axial view, (**C**) coronal view, and (**D**) three-dimensional visualization (colors: red, air cell; orange, ossicles; yellow, tegmen; green, facial nerve; blue, antrum; pink, sigmoid sinus; purple, semicircular canal and cochlea).

The scanned images of multiple detector CT (Discovery CT750 HD, GE Medical Systems, USA) were taken from a 54-year-old female patient who was scheduled for a mastoidectomy. Anonymized CT images with a 0.625 mm slice thickness were acquired. The anatomical structures required for mastoidectomy were segmented using Mimics (v. 17; Materialize Inc., Leuven, Belgium) into nonpathology areas for educational purposes. Hard tissues, such as the temporal bone, tegmen, and air cell, were segmented using a thresholding function (226 to 2873, 7 to 1002, and − 1024 to 2821 Hounsfield Units (HU), respectively) and were manually corrected by a medical 3D printing expert. Internal anatomic structures, such as the ossicles, sigmoid sinus, semicircular canal, cochlea, and facial nerve, were also segmented using a thresholding function (− 47 to 2873, 7 to 1002, 226 to 2873, 226 to 2873, and 226 to 2873 HU, respectively) and were manually corrected in the same way. Finally, the antrum was segmented using the thresholding function (− 1024 to − 35 HU) and the region from seeds manually chosen by a medical 3D printing expert, and all segmented anatomical structures were confirmed by an otolaryngologist (Fig. 2).

### Measurement of screw insertion torque

**Figure 3:**
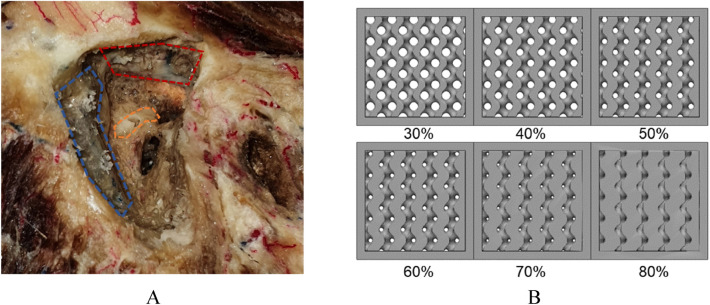
Specimens of the maximum screw insertion torque measurement. (**A**) cadaveric temporal bone with sigmoid sinus (blue), tegmen (red), semicircular canal (yellow). (**B**) 3D-printed infill specimens.

To evaluate the drilling feedback of the bone near the anatomical structures present in the temporal area, screw insertion torque was measured. An otolaryngology faculty measured screw insertion torques in nonpathological cadaveric temporal bones and box shape specimens. Four nonpathological cadaveric temporal bones were obtained and the sigmoid sinus, tegmen, and semicircular canal, screw insertion torques were measured 2 times, 2 times, and 1 time (Fig. 3A). Box shape specimens of 15 × 15 × 20 mm size were designed in the infills of 10% increments from 30 to 80%, and five specimens were fabricated for each condition using a stereolithography apparatus (SLA; Form3, Formlabs, Massachusetts, USA). with a white resin and post-cured at 60 °C for 30 min using a curing machine (Form Cure, Formlabs, Massachusetts, USA; Fig. 3B). Pilot holes were created on the sigmoid sinus, tegmen, and semicircular canal of cadaveric temporal bones until the perforation using a 1.5 mm twist drill bit made from cobalt steel (Jungpoong, Chun Cheon, South Korea).Galvanized tapping screws with a 2 mm outer diameter, 1.5 mm core diameter, 1 mm thread pitch, and 8 mm length (Bolt outlet, Incheon, South Korea) were inserted into each anatomical structure and specimen until broken. Insertion torques were measured using a digital screwdriver (Model DID-4, Imada, Northbrook, IL, USA).

### Modeling the simulator

Realistic mastoidectomy simulators were modeled using 3-matic (v. 9; Materialize Inc., Leuven, Belgium) based on the CT images. Each anatomical structure possessing unique mechanical properties offers distinct haptic feedback of drilling during surgery. Hence, each anatomical structure of the mastoidectomy simulator was modeled using different infill.

Furthermore, the average thickness of the cortical bone is approximately 1.8 mm^15^. The simulator’s cortical bone was modeling a thickness of 2 mm from the outer border to the inside and a density of 100% based on the segmented temporal bone. Moreover, internal anatomical structures, such as the facial nerve, sigmoid sinus, semicircular canal, and cochlea, were encased in bone. Each internal anatomical structure was offset with an external thickness of 2 mm to achieve a shape that protects those internal anatomical structures by being surrounded by bone. The air cell area was set entire area excluding designated cortical bone and offset area internal anatomical structures. The offset regions for the sigmoid sinus, semicircular canal, facial nerve, and cochlea were modeled gyroid structure with infill of 50%, 70%, 50%, and 70% using volumetric lattice function with Fusion 360 version 2.0.17457 (Autodesk, Inc., San Rafael, CA, USA). The original regions of each anatomical structure were modeled with the entire filled. In the same way, the tegmen, ossicles, and air cell were modeled with infill of 30%, 100%, and 20%.

**Figure 4:**
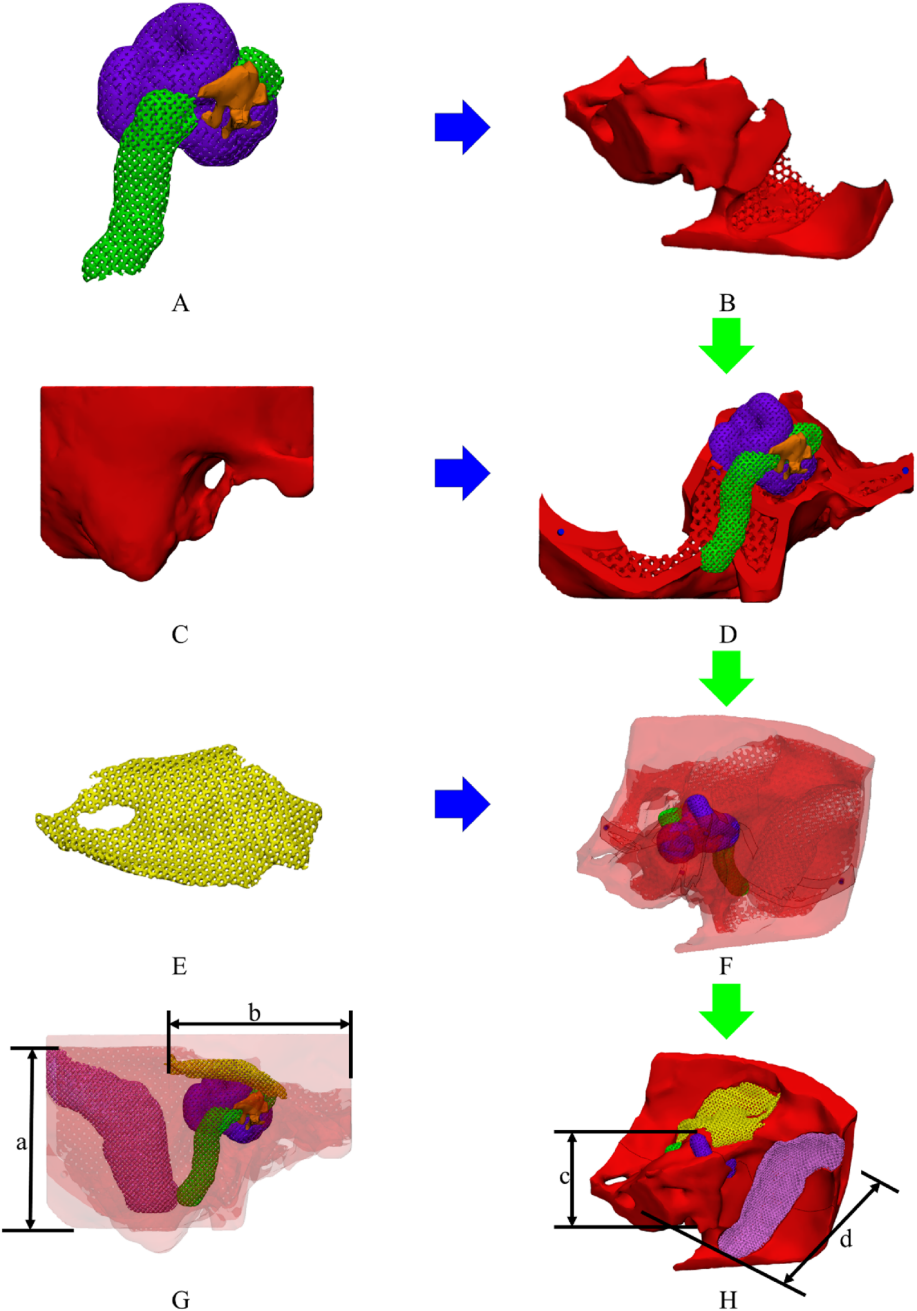
Visualization and assembly of the mastoidectomy simulator. (**A**) Semicircular canal (purple), cochlea (purple), facial nerve (green), and ossicles (yellow), (**B**) rear temporal bone, (**C**) front temporal bone, (**D**) rear temporal bone with semicircular canal, cochlea, facial nerve, and ossicles, (**E**) sigmoid sinus (pink) and tegmen (yellow), (**F**) rear temporal bone with the semicircular canal, cochlea, facial nerve, and ossicles, (**G**) front view of the transparent final mastoidectomy simulator, (a) distance between the sigmoid sinus and bottom of the temporal bone, (b) distance between the tegmen and side of the temporal bone, and (**H**) rear-view final mastoidectomy simulator, (c) distance between the semicircular canal and bottom of the temporal bone, (d) distance between the front and rear temporal bone (green arrow, assembly procedures; blue arrow, assembly spot).

The otolaryngologist empirically determined the infill of each anatomical structure based on the measured screw insertion torques of cadaveric temporal bones and 3D-printed specimens. The semicircular canal, cochlea, facial nerve, and ossicles were modeled as a single part due to the overlapping offset regions and easy attachment (Fig. 4A). The tegmen and air cell are one area with bone; thus, they were combined with the temporal bone. To assemble the internal anatomical structures, such as the facial nerve, semicircular canal, sigmoid sinus, tegmen, and cochlea, the temporal bones were divided into front and rear parts and were modeled to combine through connecting holes (Fig. 4B,C, and D).

### Fabricating the simulator

**Figure 5:**
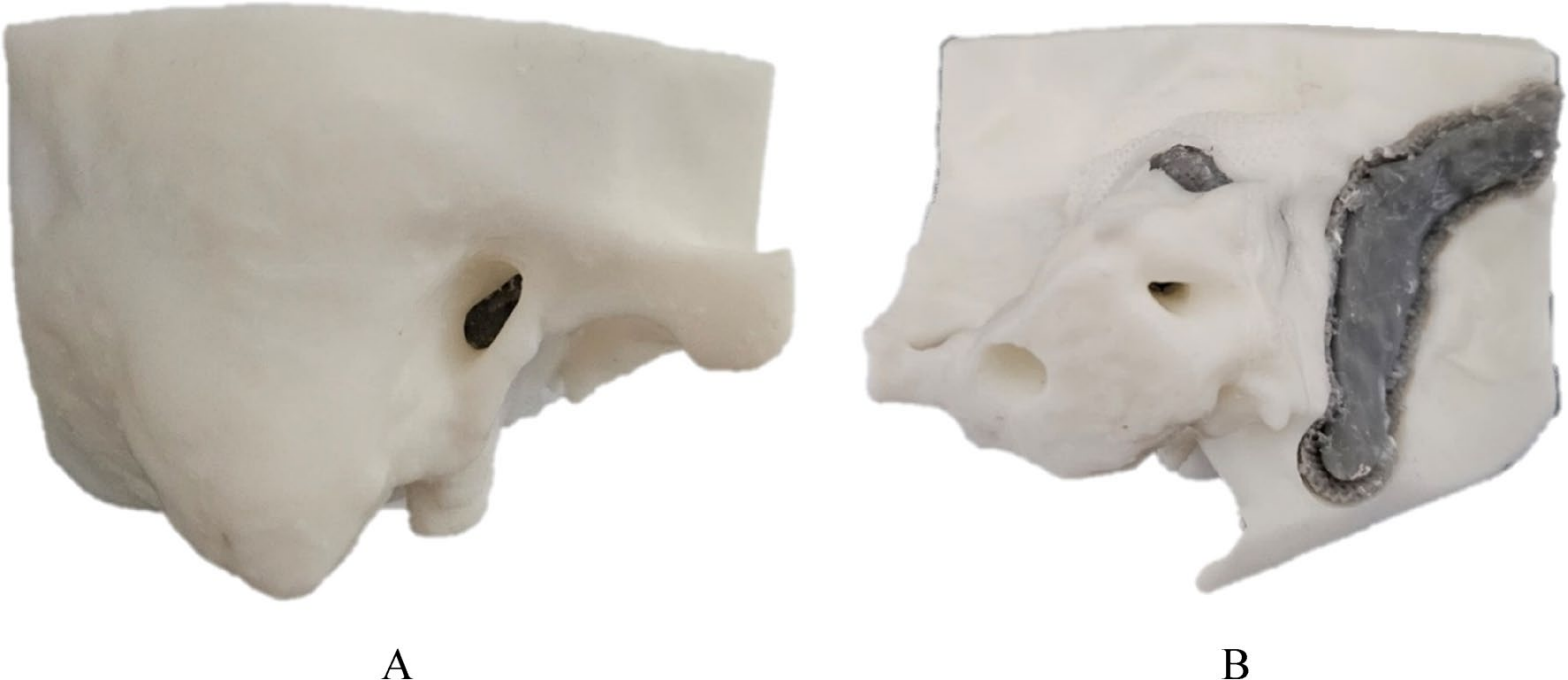
Fabricated mastoidectomy simulator considering mechanical properties and visually recognizing the difference between structures: (**A**) front view and (**B**) rear view.

All structures of the mastoidectomy simulator were fabricated using an SLA. The temporal bone, air cell, and tegmen parts were fabricated using the SLA with white resin because of their similar color to natural bone (Fig. 5A,B). The semicircular canal, cochlea, facial nerve, sigmoid sinus, and ossicles were fabricated using SLA with gray resin so the trainee could visually recognize the difference between each structure (Fig. 5B). In the study by McMillan et al. white resin received the most positive feedback among various 3D printing materials as a temporal bone simulator, and based on this, white resin was selected^12^. About 60 mL of resin was used to fabricate all parts of the temporal bone simulator, which took about 12 h, and the cost was $15 excluding labor costs.

Individually fabricated anatomical parts were assembled in three steps. First, the semicircular canal, cochlea, facial nerve, and ossicles were inserted into the rear temporal bone part and combined with the front bone part (Fig. 4A,B,D,F). Second, to bond the front and rear bone parts, a thin resin layer was applied and irradiated with ultraviolet (UV) light at the assembled bone using a UV Light Pen (3Dmon, Seoul, South Korea). Third, to bond the tegmen and sigmoid sinus, the bone parts were processed similarly to the second step (Fig. 4E,F,H). The final mastoidectomy simulator was post-cured at 60 °C for 30 min using a curing machine to enhance the mechanical properties and improve the adhesion of the assembled layer^16^.

### Shape accuracy

Accurately positioning the internal anatomical structures can further enhance the training effects for the trainee. The simulator was fabricated in five parts and combined into one part using internal grooves and holes. Therefore, we evaluated whether the shape changed during fabrication and post-processing. Four landmarks were designated in the simulator: distance between the sigmoid sinus and body, distance between the tegmen and body, distance between the semicircular canal and body, and thickness of the body. Two medical 3D printing experts measured the distance of four landmarks of the stereolithography models and fabricated the parts five times using 3‑Matics and Vernier calipers with a repeat accuracy of 0.01 mm (CD-30AX, Mitutoyo Co., Japan; Fig. 4G and H). A Bland–Altman analysis was used to evaluate the shape accuracy of the mastoidectomy simulator using Med-Calc (v. 19; MedCalc Software Ltd., Acacialaan, Belgium).

### Quality and validity assessment

Two otolaryngology residents and three faculty with informed consent were recruited to evaluate the quality and feasibility of the fabricated mastoidectomy simulator. Each participant drilled the simulator without a time limit at their own pace and scored the quality, validity, and educational value assessment for the fabricated mastoidectomy simulator on a five-point Likert scale. The first questionnaire asked about the quality of the simulator compared to in vivo temporal bone surgery, which included the similarity of the bone and internal anatomy (1 = dissimilar, 2 = rarely similar, 3 = slightly similar, 4 = almost similar, 5 = perfectly similar; Table 2). The second questionnaire asked about the validity and educational value of the simulator (1 = strongly disagree, 2 = disagree, 3 = partially agree, 4 = agree, 5 = strongly agree; Table 3). This study protocol for quality assessment was approved by the Institutional Review Board of Asan Medical Center (2023-0292) and informed consent from each participant was waived. Cronbach's Alpha is used to estimate Internal consistency through pingouin package in Python.

## Results

### Screw insertion torque

The otolaryngologists measured the screw insertion torque values of the cadaveric temporal bones and 3D-printed specimens. The screw insertion torque of five 3D-printed specimens for each condition and four nonpathological cadaveric temporal bones were measured. The maximum insertion torque values (N mm; mean ± standard deviation) for the sigmoid sinus, tegmen, and semicircular canal were 1.08 ± 0.62, 0.44 ± 0.42, and 1.54 ± 0.43. The sigmoid sinus, tegmen, and semicircular canal displayed a similar insertion torque to the specimens’ infill of 40%, 30%, and 50% (Table 1). This study protocol for quality assessment was approved by the Institutional Review Board of Asan Medical Center (2023-0292) and informed consent from each participant was waived.

**Table 1:** Maximum screw insertion torque (N mm) of the three-dimensional-printed specimens with different infill and cadaveric anatomies.

Specimens	*N*	Mean	SD
30%	5	0.66	0.22
40%	5	1.10	0.08
50%	5	1.21	0.04
60%	5	1.95	0.21
70%	5	2.14	0.17
80%	5	3.25	0.11
Sigmoid sinus	8	1.08	0.62
Tegmen	8	0.44	0.42
Semicircular canal	4	1.54	0.43

### Shape accuracy

**Figure 6:**
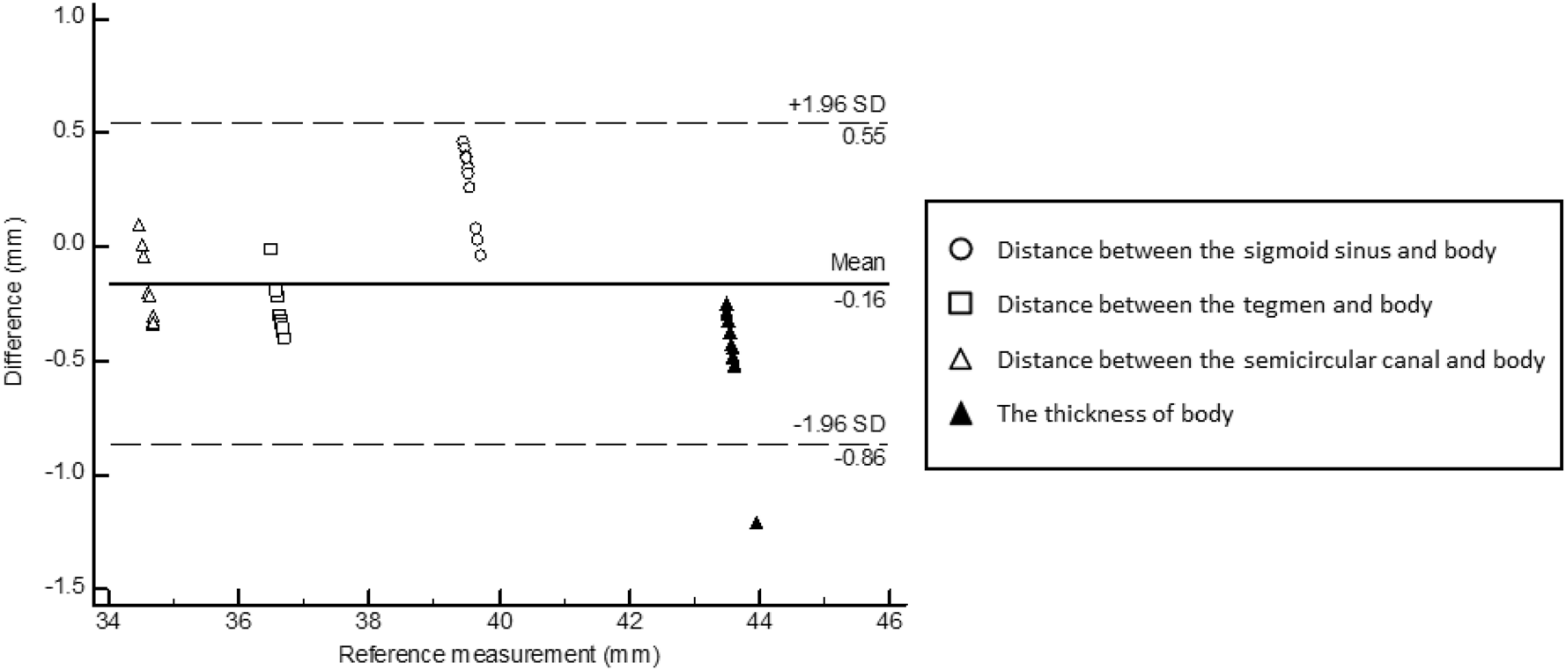
Bland–Altman plot evaluating differences between the stereolithography model and assembled simulator.

Two medical 3D printing expert measured the distance between the internal anatomical structure and the simulator body of the stereolithography models and fabricated the simulator five times. A total of 40 landmarks were evaluated using the Bland–Altman analysis. The measurement error (mean ± SD) was − 0.16 ± 0.32 mm (limit of agreement − 0.86 to 0.55; Fig. 6). All measurements, except for the body thickness, were within the 95% limits of agreement.

### Quality and usability assessment

**Figure 7:**
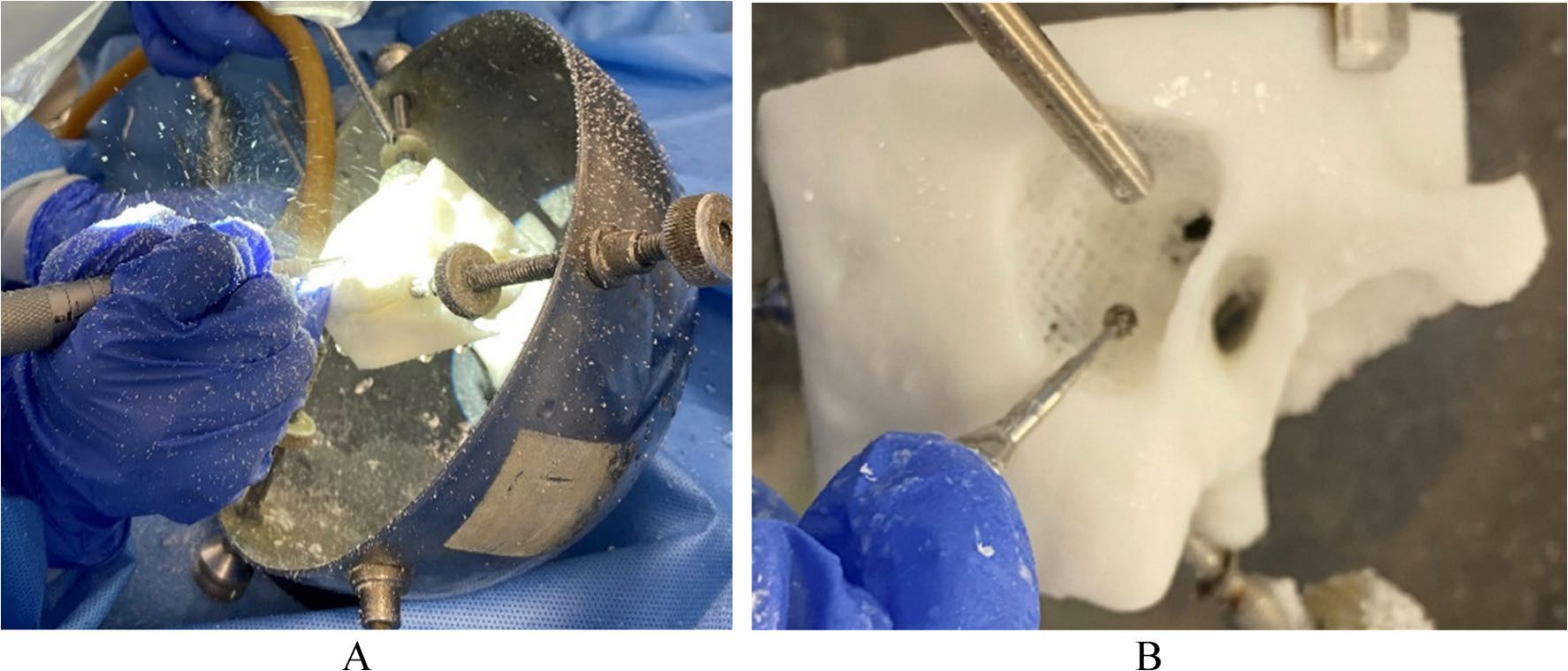
Simulation of the mastoidectomy simulator considering mechanical properties. (**A**) The mastoidectomy simulator was drilled while fixed on a jig. (**B**) Front view of the mastoidectomy simulator with the air cell removed.

Experienced mastoidectomy two otolaryngology residents and three otolaryngology faculty members drilled and assessed a mastoidectomy simulator (Fig. 7). Likert scale survey about quality and validity was evaluated by comparing the participants' experience drilling live surgery.

The simulator received favorable quality scores, with the similarity of each anatomical structure and the drilling texture of the cortical bone scoring a median of 5 and an interquartile range (IQR) of 0.5. However, the structure surrounding the facial nerve was rated lower, with a median of 3.0 and IQR of 2 (Table 2).

**Table 2:** Quality assessment of the fabricated simulator using a five-point Likert scale (1 = dissimilar, 5 = similar).

List	Median (IQR)
The similarity of the cortical bone	5 (0.5)
The similarity of the air cell and tegmen	4 (1)
The similarity of the structure around the sigmoid sinus	5 (1)
The similarity of the structure around the semicircular canal and cochlea	4 (1.5)
The similarity of the structure around the facial nerve	3 (2)
The similarity of the positions and shapes of the overall anatomical structure	5 (1)
The similarity of the overall drilling texture	4 (1.5)

Moreover, the mastoidectomy simulator was highly usable with educational value, as suggested by the response questionnaire. All following aspects of the simulator median of 4 or higher and were commended: facilitating the understanding of temporal bone anatomy, improving surgical skills, boosting confidence in surgery, resemblance to the actual clinical environment, the appropriate level of difficulty for a training environment, and the likelihood of recommending this simulator to other trainees (Table 3). Cronbach's alpha about quality assessment and usability assessment is 0.87 and 0.77.

**Table 3:** Usability and educational value assessment of fabricated simulator using a five-point Likert scale (1 = disagree, 5 = agree).

List	Median (IQR)
This simulator helped understand temporal bone anatomy	5 (0.5)
This simulator helped improve surgery skills	5 (1)
This simulator was similar to the actual clinical environment	5 (0.5)
This simulator provided the appropriate level of difficulty for the training environment	4 (1)
This simulator improved confidence for surgery	4 (1)
Recommend this simulator to other trainees	5 (1)

## Discussion

We developed a mastoidectomy simulator using 3D printing based on CT images that reproduced the haptic feedback of the drilling of each anatomical structure by designing different infill. Moreover, it enhanced the educational utility regarding the bone and other anatomical parts using assorted colors and demonstrated a realistic simulator that enabled functional tasks during mastoidectomy training. Our mastoidectomy simulator was fabricated using SLA, which is an affordable method compared to commercial temporal bone phantom (PHACON GmbH, Leipzig, Germany) fabricated based on color-jet printing. The low fabrication cost of $15 suggests the high accessibility of education using simulators. Moreover, the white resin used to fabricate simulators, a thermoset polymer, does not change due to heat generated by drilling, and these characteristics make them suitable for use in simulators compared to thermoplastics, which are generally used in 3D printers^17^. The development of the mastoidectomy simulator is primarily aimed at enhancing the training of novices with no prior experience in mastoidectomy. Our mastoidectomy simulator holds significant promise for medical education, facilitating the instruction of trainees on the intricacies of managing complex anatomical variations and pathologies, such as those encountered in post-traumatic complications and cholesteatomas through anatomic-specific modeling. Furthermore, the utilization of mastoidectomy models extends beyond educational purposes; they serve as valuable tools in preoperative planning, enabling surgeons to assess the feasibility of surgical interventions and to make informed decisions regarding surgical indications. This approach not only augments the educational framework for surgical trainees but also contributes to the optimization of patient care by allowing for personalized surgical strategy and planning. Existing studies on temporal bone simulators using 3D printing evaluated the quality and usability of the simulator relatively subjectively through surveys. A strength of our study is that we objectively evaluated the simulator through a comparison of screw insertion torques in cadaveric temporal bones and 3D-printed specimens^9,11–13^.

The otolaryngologists determined the infill structures of the semicircular canal, facial nerve, cochlea, tegmen, ossicles, and air cell using empirical judgment based on the measured screw insertion torques of the cadaveric temporal bones and 3D-printed specimens, leading to an overall favorable quality assessment of the mastoidectomy simulator. Furthermore, the structure surrounding the sigmoid sinus, semicircular canal, and cochlea received high scores overall. However, the structure surrounding the facial nerve scored relatively low (3.40 ± 1.14). In the structure surrounding the facial nerve, otolaryngology residents provided high scores; however, the otolaryngology faculty assigned relatively low scores. The facial nerve is surrounded by a bony canal known as the fallopian canal. In reality, this nerve, which has a pinkish appearance, is not initially visible due to the surrounding bone. However, as the bone gradually thins through drilling, the facial nerve is progressively exposed and takes on the appearance of a pinkish nerve structure. In other words, as the bone covering the facial nerve becomes thinner, its location and shape become more visible. It is predicted that the fabricated simulator may receive a low score due to limitations in reproducing this gradual bony thinning process accurately with the change of color. We also assumed that the relatively thin structure of the facial nerve had a stronger drilling reaction force than expected because the resin was trapped in the process of fabricating^18^. The positions and shapes of the overall anatomical structure received high scores because the mastoidectomy simulator was fabricated based on CT images.

Furthermore, the overall shape of the fabricated simulator exhibited reasonable errors (limit of agreement − 0.86 to 0.55 mm) using the Bland–Altman plot. Although the error was slightly high in measurements of the body thickness, there seems to be no problem in use, as the difference was within 1.2 mm. Moreover, the simulator received a favorable validity and educational value assessment. Understanding the temporal bone anatomy, similarity to the actual clinical environment, confidence for surgery after training, improving surgery skills, and recommending this simulator to other trainees received high scores.

However, the difficulty level for the training environment received a relatively low score (4.00 ± 0.70). The mastoidectomy simulator was fabricated for nonpathological areas for educational purposes. In the case of a patient with chronic otitis media, the mastoid is often more sclerotic, unlike the fabricated simulator we drilled out. Drilling out a sclerotic mastoid can indeed pose greater difficulty in identifying the antrum, and it can also make distinguishing it from surrounding structures more challenging; thus, the difficulty level for the training environment received a relatively low score for faculty members. The cost of materials for fabricating the simulator was modest, suggesting increased accessibility and opportunities for repeated training compared to using cadaveric temporal bones and high-fidelity models. Moreover, drilling a cadaveric temporal bone for a simple mastoidectomy training process may be more customized and anatomically accurate when using 3D printing. This allows for the replication of the unique anatomical features specific to each individual patient, enhancing the practice experience. These results demonstrate that the simulator could be used for educational purposes for mastoidectomy training with significant enhancement.

Despite these promising results, this study has limitations. First, while the simulator was designed to mimic the mechanical properties of bones adjacent to the internal anatomy, it was not possible to replicate the mechanical properties of each anatomical structure. Consequently, the simulator may not provide an accurate representation of the sensation that would be experienced if the internal anatomy was accidentally damaged during a mastoidectomy. However, training on the simulator does allow for the development of a sense of the resection of bones adjacent to the internal anatomy, which is helpful for surgical training. In addition, to address this, we plan to fabricate and evaluate a temporal bone simulator that more realistically mimics the mechanical properties of cancellous and internal anatomy based on evaluating the screw insertion torque of a variety of 3D printable materials. Second, the simulator was not extensively evaluated by multiple groups. To address this, we plan to conduct a multicenter study to evaluate the simulator’s effectiveness and identify opportunities for further improvement and enhancement for educational purposes. Third, the proposed model was not compared to commercial educational models. In our subsequent study, we plan to supplement the proposed model with various comparison groups, such as animal models and commercial models, to accurately evaluate its performance. Fourth, the temporal bone simulator was modeled the simulator using the offset function to simplify the modeling process, and these approaches caused a decrease in the visual quality. In future research, we will simplify the modeling process through accurate segmentation of anatomical structures using artificial intelligence to reduce labor time and increase visual quality. This approach is closer to anatomic-specific modeling than current approaches. Fifth, the temporal bone simulator was fabricated based on images with relatively thin slice thickness (0.6 mm)^19^. In general, capturing 3D trabecular bone shapes (less than 100 μm) of diseases is not easy due to modern CT scan limitation such as motion blur, partial volume, gantry vibration, etc.^20^. We will complement the value by creating a simulator using high-resolution images with recent photon counting CT or more advanced CT techniques. Sixth, we were unable to directly measure the drilling reaction force of 3D-printed specimens and cadavers. We will construct a system that can directly measure the drilling reaction force (a jig for fixing the 3D printing specimens and cadavers, a load cell for measuring reaction force, and a motor for rotating the burr) to measure the drilling reaction force of more various 3D printable materials and cadavers. We will also fabricate and evaluate a more realistic simulator by fabricating a simulator based on measured drilling reaction force. Finally, reproducibility for various disease models was lacking. We plan to fabricate various disease models for improvement and enhancement for educational purposes.

## Conclusion

We aimed to improve surgeon skills and improve patient health by providing a realistic simulation of a mastoidectomy. We successfully developed a mastoidectomy simulator to provide a realistic drilling sensation of bone by modeling different infill of each anatomical part and enhancing education using assorted colors for bone and other anatomical parts. The realistic training environment provided by the mastoidectomy simulator may reduce potential risks to patients by improving surgeon skills.

## Data Availability

The generated datasets in this study are not publicly available because the data were created based on patient images but are available from the corresponding author on a reasonable request.
